# Supracerebellar transtentorial resection of a ruptured thalamomesencephalic cavernous malformation

**DOI:** 10.3171/2019.7.FocusVid.19164

**Published:** 2019-07-01

**Authors:** Michel W. Bojanowski, Moujahed Labidi, Nathalie L’Ecuyer, Chiraz Chaalala

**Affiliations:** Division of Neurosurgery, Department of Surgery, Centre Hospitalier de L’Université de Montreal, Quebec, Canada

**Keywords:** cavernous malformation, resection, thalamus, brainstem, supracerebellar transtentorial, video

## Abstract

Thalamomesencephalic cavernous malformations are located high in the brainstem and may be difficult to reach. We present a case of such a lesion which was successfully approached via the supracerebellar transtentorial route. Our enclosed video provides elements to justify this posterior approach and illustrates the steps required for the cavernoma’s safe removal, which include opening of the tentorium and gentle retraction of the exposed temporal lobe.

The video can be found here: https://youtu.be/Ex5OfLyBzPY.

## Transcript

My name is Michel Bojanowski and with this video I would like to illustrate the steps required to remove a thalamomesencephalic cavernoma.

This 36-year-old woman, without previous medical history, presented with a sudden onset of right hemiplegia associated with diplopia, speech impediments, and drowsiness. The head CT scan revealed a left thalamic hematoma extending into the upper part of the midbrain; we suspected the rupture of a thalamomesencephalic cavernoma. A few hours later, the patient became very lethargic. A second CT scan showed a mild increase of the hematoma, as well as hydrocephalus. The level of consciousness improved following a ventriculostomy, but the other symptoms remained unchanged. MRI also suggested that the bleeding was due to a cavernous malformation, and that the bleeding reached the anterior surface of the brainstem.

Because of the severity of the motor and speech deficits due to the hematoma’s large size, we proposed to proceed at the first available opportunity, which happened to be on the sixth day following admission (Garcia et al., 2015).

Despite the fact that the hematoma reached the anterior surface of the brainstem and that therefore it could be drained anteriorly without transgressing the brainstem via an orbitozygomatic craniotomy (Rangel-Castilla and Spetzler, 2015), we opted for a posterolateral approach. Our reasons were that the lateral mesencephalic sulcus safe entry zone (Giliberto et al., 2010; Asaad et al., 2010; Abla et al., 2010) was close to the hematoma, that this approach allows a greater upward angle towards the thalamus, access is less cumbersome regarding the perforators, and the route is shorter.

In park bench position, with the left side up, head flexed and elevated for venous drainage, a 5-cm left paramedian skin incision is made to expose the occipital bone. A suboccipital craniotomy is performed, extending upward to expose the inferior portion of the transverse sinus, and laterally to expose the medial border of the sigmoid sinus. The size of the craniotomy should aim to limit as much as possible cerebellar retraction. The dura is opened in a triangular shape with the base at the transverse sinus.

Using the infratentorial supracerebellar approach, the cerebellomesencephalic cistern is exposed, benefiting from the space created by the pull of gravity. Through the arachnoid, we can clearly see the fourth cranial nerve.

The neuronavigator shows us that the surgical target lies hidden within the brainstem above the level of the tentorium and above the trochlear nerve.

Therefore, we incise the tentorium in a posteroanterior straight line.

The temporal lobe thus exposed needs to be gently retracted upward. In order to do so, the arachnoid of the cerebellomesencephalic cistern must be incised.

Using sutures, the tentorial flaps are folded back to increase exposure.

The arachnoid is further incised to reduce the amount of force necessary to gently retract the temporal lobe.

Since we do not see the lesion at the surface of the brainstem, the point of entry is determined by the neuronavigator at the lateral mesencephalic fissure.

After a sharp incision with a beaver blade is made at the surface of the brainstem, without any coagulation, gentle retraction of the white matter allows us to reach the cavernoma.

Opening the hematogenic wall allows the collected fluid blood to drain. Now with the brainstem relaxed, we can see the normal pulsation.

The soft intracapsular component is debulked using suction and biopsy tweezers.

Extracapsular dissection is performed using microscissors for sharp dissection when needed.

To remove the cavernoma, the working window inside the brainstem gets gradually stretched as we work. The cavernoma is gently pulled out respecting the natural pulsation of the brain and the viscoelasticity of the tissues.

The bed of the cavernoma is explored to ensure complete resection. Hemostasis is ensured. The opening in the brainstem is covered with Surgicel.

The leaflets of the tentorium are brought back.

Following watertight closure of the dura and repositioning of the bone flap, closure is done in the usual fashion.

This is the CT scan the day after the surgery.

In less than 1 week following surgery, the patient was able to lift her affected leg and to move her fingers.

MRI showed complete resection of the lesion.

### Key points regarding this case:

• Exposure requires opening the tentorium and lifting the temporal lobe.
• Neuronavigator is essential to determine the point of entry in the brainstem.
• Neuronavigator is helpful to determine where to open the tentorium.
• Reaching the cavernoma is best with the smallest possible opening in the brainstem, which is then enlarged as we work.
• For large lesions, debulking is first, followed by extracapsular dissection.
• Pulling the cavernoma out is done gently going along, when possible, with the natural pulsation of the brain and respecting the viscoelasticity of the tissues.

## Time points

1:38 Justification of choice of approach

2:53 Exposure of the cerebellomesencephalic cistern

3:23 Incision of the tentorium

3:48 Arachnoid incision of the cerebellomesencephalic cistern

4:00 Retraction of the tentorial flaps

4:30 Use of the neuronavigator for safe entry zone

4:40 Incision of the brainstem

4:48 Exposure of the cavernous malformation

5:12 Intracapsular resection

5:49 Pulling out the cavernoma

8:07 Key points
